# Lower myocardial stress perfusion in infarct-adjacent than in remote myocardium four months after revascularized myocardial infarction

**DOI:** 10.1186/1532-429X-17-S1-P123

**Published:** 2015-02-03

**Authors:** Kaatje Goetschalckx, Frank E Rademakers, Jan Bogaert, Attila Toth, Béla Merkely, Stefan Janssens, Piet Claus

**Affiliations:** Cardiovascular Diseases, University Hospital Leuven, Leuven, Belgium; Department of Cardiovascular Sciences, KU Leuven, Leuven, Belgium; Department of Radiology, University Hospitals Leuven, Leuven, Belgium; Heart and Vascular Center, Semmelweis University, Budapest, Hungary

## Background

Myocardial infarction (MI) leads to heart failure in a substantial number of patients. Investigation of temporal dynamics of perfusion in the infarct-adjacent segments can offer better insights into left ventricular remodeling. It is the aim of this study to compare myocardial blood flow (MBF) with cardiac magnetic resonance (CMR) in the infarct borderzones (BZ) and the remote myocardium (RM) in a large patient population 4 months after MI.

## Methods

In this substudy of the NOMI-trial (ClinicalTrials.gov identifier: NCT01398384) using the placebo arm, 64 patients underwent a CMR with rest and adenosine stress perfusion 4 months after acutely revascularized ST-elevation MI, in a 1.5 tesla MR scanner (Achieva, Philips Medical Systems, The Netherlands). TIMI 2-3 flow was achieved in all patients after PCI. MBF was quantified using Fermi deconvolution with a dual bolus analysis technique (equal volumes of 0.0027 mmol/kg followed by 0.05 mmol/kg after a 20-s pause of contrast agent (Dotarem, Gd-DOTA, Guerbet, France)) in basal and midventricular short axis perfusion slices. For stress imaging, 140 µg/kg/min of adenosine was administered intravenously for approximately 4 minutes. Stress and rest imaging were separated by at least 10 minutes. A segmental model was applied on corresponding images of perfusion and late gadolinium enhancement (LGE). The MBF of the infarct zone (IZ) was calculated as the mean perfusion of segments with LGE. The mean perfusion of the adjacent 30 degree segments was defined as MBF of the BZ. The mean perfusion of the remaining segments determined the MBF of the RM. Apical slices seldomly included more than one myocardial zone and were excluded from analysis.

## Results

One hundred twenty eight slices were analyzed for rest and stress perfusion. Rest perfusion 4 months after MI was not significantly different in BZ and RM (resp. 0.42 ± 0.20 and 0.44 ± 0.20 ml/g/min, p = 0.262) . Stress perfusion however, was significantly lower in BZ than in RM (resp. 1.16 ±.53 and 1.32 ±.57 ml/g/min, p < 0.05). (cf. Table)

## Conclusions

Myocardial stress perfusion in infarct-adjacent segments is significantly impaired 4 months after revascularized MI. Persistend regional perfusion deficit may offer new opportunities for targeted angiogenic therapies.

## Funding

No disclosures.

**Figure 1:**
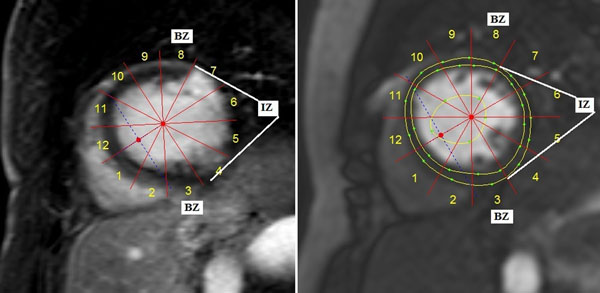
The left figure shows the late gadolinium enhancement midventricular short axis slice of an MI revascularized 4 months earlier, with segments 4-7 defined as IZ, segments 3 and 8 as BZ and the remaining segments as RM. The right figure shows the corresponding restperfusion slice.

**Figure:**
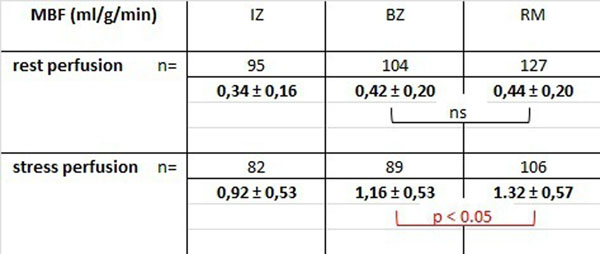
Figure 2

